# Laser injury promotes migration and integration of retinal progenitor cells into host retina

**Published:** 2010-06-04

**Authors:** Caihui Jiang, Henry Klassen, Xinmei Zhang, Michael Young

**Affiliations:** 1 Schepens Eye Research Institute, Department of Ophthalmology, Harvard Medical School, Boston, MA; 2 Department of Ophthalmology, Chinese Military General Hospital, Beijing, China; 3 Department of Ophthalmology, School of Medicine, University of California, Irvine, Orange, CA

## Abstract

**Purpose:**

The migration and integration of grafted cells into diseased host tissue remains a critical challenge, particularly in the field of retinal progenitor cell (RPC) transplantation. It seems that natural physical barriers at the outer retina can impede the migration of grafted RPCs into the host retina. The purpose of this study was to investigate the integration and differentiation of murine RPCs transplanted into the subretinal space of mice with laser-induced damage to the outer retina.

**Methods:**

RPCs were harvested from the neural retinas of postnatal day 1 enhanced green fluorescent protein (GFP) mice. Retinal photocoagulation was performed using a diode laser. Two µl containing ~6×10^5^ expanded RPCs in suspension were injected into the subretinal space of the recipient animals following laser treatment. Cell morphometry was performed to assess the integration of donor cells. Immunohistochemistry and western blot were performed on recipient retinas.

**Results:**

Three weeks after transplantation, 1,158±320 cells per eye had migrated into the recipient outer nuclear layer (ONL). Most of these cells resided in the ONL around the retinal laser lesion. A subpopulation of these cells developed morphological features reminiscent of mature photoreceptors, expressed photoreceptor specific proteins including synaptic protein, and appeared to form synaptic connections with bipolar neurons. Retinal photocoagulation resulted in a significantly increased expression of matrix metalloproteinase-2 (MMP-2), MMP-9, and cluster differentiation 44 (CD44s), and a decreased expression of neurocan.

**Conclusions:**

Transplanted RPCs migrate and integrate into the laser-injured ONL where they differentiate into photoreceptors with morphological features reminiscent of mature photoreceptors, express synaptic protein, and appear to form synaptic connections with retinal bipolar neurons. Following retinal photocoagulation, the enhanced level of integration of grafted RPCs is partially associated with increased expression of MMP-2 and, to a lesser extent, MMP-9, together with decreased levels of inhibitory molecules.

## Introduction

Millions of people worldwide suffer visual disabilities as a result of retinal diseases that lead to the loss of photoreceptors. Due to the lack of effective central nervous system (CNS) regeneration, this photoreceptor loss is irreversible. Repair of such damage by cell replacement is one promising treatment strategy for retinal degenerative diseases. The possibility of using stem cell transplantation to treat retinal degenerative diseases has triggered enthusiasm, and much hope has been placed on the potential use of these cells to restore visual function. However, there are critical challenges facing retinal cell transplantation, such as cell delivery and the limited survival, integration, and control over differentiation of grafted cells [1,2]. Although a recent study showed that post-mitotic photoreceptors can achieve wide-spread integration, the source of a sufficient number of such cells remains problematic [3]. So far, retinal progenitor cells (RPCs) and other stem cells transplanted into various adult animal models of retinal degeneration have shown only limited evidence of being able to integrate into the outer nuclear layer (ONL) and differentiate into new photoreceptors [4–7]. Among factors that may contribute to limited donor cell integration are natural physical barriers that impede the migration of immature neurons and progenitor cells [8–10]. In any case, higher levels of integrated grafted cells are needed to improve or restore vision.

It is well known that the regenerative capacity of the adult mammalian CNS is extremely limited with respect to both the generation of new neurons and the reconstruction of synaptic pathways. This inability to regenerate can be attributed to the inhibitory extracellular matrix (ECM) and cell adhesion molecules. Previous studies have shown that inhibitory chondroitin sulfate proteoglycans (CSPGs) such as neurocan and glycoprotein CD44 are abundant in CNS, and these molecules have also been proven to function as chemical inhibitors of neurites and axonal growth, preventing functional regeneration of several different cell types. In addition, inhibition of these molecules has been shown to enhance axonal regeneration [11–14].

Matrix metalloproteinase (MMPs) are key processing enzymes to degrade ECM and cell adhesion molecules to modulate cell behavior. Among the substrates targeted by MMPs, the inhibitory CSPGs and CD44 are included [15–18]. For example, MMP-2 and MMP-9 have been shown to degrade CSPGs (neurocan), increasing the cell motility and neurite outgrowth for laminin-induced neurite outgrowth from peripheral sensory neurons [15].

As part of the CNS, both normal and degenerative retina express neurocan and CD44 [19–23]. The presence of these inhibitory molecules—neurocan and CD44—at the outer surface of the retina may impede the RPC migration and integration, hindering successful RPC transplantation [22,23]. A recent study also suggests that the outer limiting membrane (OLM) presents a physical barrier to photoreceptor integration following transplantation into the subretinal space in adult mice; disruption of the OLM leads to increased retinal integration of transplanted photoreceptor precursors [24].

In a previous study, we have shown that CSPGs, neurocan and glycoprotein CD44, pose a barrier to the extension of neurites and cell integration. And adding activated recombinant MMP-2 to explant cultures results in the proteolysis of neurocan and CD44 in the degenerative retina, allowing increased outgrowth across the border between abutting nondystrophic and rd1 retinas in vitro [23].

Laser-induced damage mainly involves the outer layers of the retina where energy is absorbed by the pigmented cells of the retinal pigment epithelium (RPE) and then transferred as heat to the adjacent outer layers [25]. In the present study, we sought to specifically investigate the integration and phenotypic differentiation of murine green fluorescent protein (GFP)-positive RPCs transplanted into the subretinal space in mice with laser-induced damage to the outer retina.

## Methods

### Animals

All experiments were performed in compliance with the ARVO Statement for the Use of Animals in Ophthalmic and Vision Research, and all experimental protocols were approved by the Animal Care and Use Committee of the Schepens Eye Research Institute. C57BL/6J (B6) mice (8 weeks, Charles River Laboratories) were housed in a 12 h light-dark cycle with water and food provided ad libitum.

### Cell isolation and culture

Retinal progenitor cells harvested from the neural retinas of postnatal day 1 enhanced green fluorescent protein mice (C57BL/6 background; a gift from Dr. Okabe, University of Osaka, Osaka, Japan) were isolated and maintained in culture as previously described [26]. Briefly, neural retinas were dissected free from surrounding tissues. Pooled neural retinal tissue was minced and dissociated enzymatically (0.1% collagenase in Hank’s buffered salt solution [HBSS], Sigma, St. Louis, MO) at room temperature. Liberated cells were collected through a 100 µm mesh strainer, centrifuged, and then resuspended in neurobasal medium which was supplemented with epidermal growth factor (EGF, 20 ng/ml), L-glutamine (2 mM, Sigma), nystatin (2,000 U, Sigma), penicillin/streptomycin (100 µg/ml, Sigma), and 2% B-27 supplement and N-2 supplement (Invitrogen, Carlsbad, CA). Subsequently, cells were fed by 50% medium exchange every 2–3 days and passaged at confluence using Cell Dissociation Buffer (Gibco, Grand Island, NY), centrifugation, and gentle trituration.

### Retina photocoagulation and transplantation

B6 mice were deeply anesthetized with an intraperitoneal injection of ketamine (120 mg/kg) and xylazine (20 mg/kg), followed by the dilation of the pupils with 0.5% topical tropicamide (all from Phoenix Pharmaceutical Inc., St. Joseph, MO). Photocoagulation was performed using a diode laser (spot size: 350 µm; power: 100 mW; duration: 100 ms; 12 burns per eye).

Immediately after photocoagulation, a small, self-sealing sclerotomy was made and a glass microneedle (attached to a 50 µl Hamilton syringe via polyethylene tubing) was advanced through the sclera under direct visualization. Two microliters of fluid containing ~6×10^5^ cells of passage 5 were slowly injected into the subretinal space to produce a retinal bleb in the vicinity of the injection site. During transplantation, the intraocular pressure was temporarily reduced by making a small puncture through the cornea.

### Histology and immunohistochemistry

Tissue fixation, sectioning, and immunohistochemistry were performed as described previously [27]. Mice were sacrificed by CO_2_ inhalation 3 weeks after transplantation. For laser damage assessment, mice were sacrificed and the enucleated eyes were immediately put in 4% paraformaldehyde (PFA) in phosphate-buffered saline (PBS). The eyes were fixed in 4% PFA overnight, cryoprotected in serial sucrose solutions, frozen in optimal cutting temperature compound (Tissue-Tek, Miles Diagnostic Division, Elkhart, IN), sectioned in their entirety at 10 µm, mounted on Superfrost Plus slides (VWR Scientific, West Chester, PA), and stored at −80 °C for further study. All sections were collected for analysis. The following primary antibodies were used: rabbit anti-GFP (1:100; Clontech), mouse anti-rhodopsin (1:100; Chemicon Temecula, CA), rabbit anti-recoverin (1:1,000; Chemicon), mouse anti-bassoon (Stressgen), goat anti-protein kinase C alpha (PKCα; 1:100; Santa Cruz, CA), rabbit anti-GFAP (1:200; Invitrogen, Carlsbad CA), rabbit anti-MMP-2 (1:100; Chemicon, Temecula, CA), rabbit anti-MMP-9 (1:100; Chemicon), CD44 (1:100; BD PharMingen), and rat anti-CD44s (1:100; Chemicon). The following secondary antibodies were used: fluorescein isothiocyanate (FITC)-conjugated AffiniPure goat anti-Mouse IgG (1:200), FITC-conjugated AffiniPure goat anti-rabbit IgG (1:200), FITC-conjugated AffiniPure mouse anti-goat IgG (1:200), FITC-conjugated AffiniPure goat anti-mouse IgG (1:200), Cy3-conjugated AffiniPure donkey anti-goat IgG (1:800), Cy3-conjugated AffiniPure goat anti-mouse IgG (1:800), and Cy3-conjugated AffiniPure goat anti-rabbit IgG (1:800; all from Jackson ImmunoResearch Laboratories, Inc., West Grove, PA). Nuclei were counter-stained with To-Pro-3 (1:1,000; Invitrogen). Negative controls for immunolabeling consisted of substituting normal serum in place of the primary antibodies. Retinal sections were viewed on a confocal microscope (Leica SP2, Leica Microsystems GmbH, Wetzlar, Germany).

### Western blot

Western blot analyses were performed as described previously [28]. Three days after retinal photocoagulation, retinas (5 eyes for each group) were collected and homogenized in lysis buffer (50 mM Tris-HCl, pH 7.6, 150 mM NaCl, 10 mM CaCl_2_, and 1% Triton X-100, 0.02% NaN_3_). Supernatants were isolated and protein concentrations were determined using a BCA protein assay (n=6 [total 6x3=18 B6 mice]; Pierce Chemical, Rockford IL). Equivalent amounts of protein (50 µg) were subjected to sodium dodecyl sulfate PAGE (SDS–PAGE; 8%–10% acrylamide), transferred to nitrocellulose, and probed with the following antibodies: pro-MMP-2, pro-MMP-9 (1:1,000; Chemicon), CD44s (1:1,000; Sigma), neurocan (1:1,000; a gift from Richard Margolis, Department of Pharmacology, New York University), and β-actin (1:1,000; used as a loading control; Abcam, Cambridge, MA). Blots were cut and reprobed sequentially, visualized with ECL reagents (NEN, Boston, MA), and exposed to X-ray film (Bio Max light Film, Lodak/Carestream Health, Rochester, NY). Developed films were subsequently digitized and densitometrically analyzed with Image J software (National Institute of Heath; each substrate was normalized against β-actin). Digital images of western blots were used to make composite figures with graphics software (Adobe Photoshop; Adobe Systems Inc., San Jose, CA).

### Cell counts

The cell counting during histology analysis was performed as described previously [3]. Three weeks after transplantation, the animals were sacrificed and the eyes were prepared for analysis as described above. Cells were considered to be integrated if the whole cell body was correctly located within the ONL and at least one of the following was visible: spherule synapse, inner/outer processes, and/or inner/outer segments. The average number of integrated cells per section was determined by counting all the integrated GFP-positive cells in every 1 in 4 serial sections through the site of injection in each eye. This was multiplied by the total number of sections that encompassed the injection site to give an estimate of the mean number of integrated cells per eye (Nine eyes were sampled per group).

### Statistical analysis

The data were expressed as means±SD (n=9). Data between groups were compared using a Student’s *t* test. Statistical significance is declared at p<0.05.

## Results

**Figure 1:**
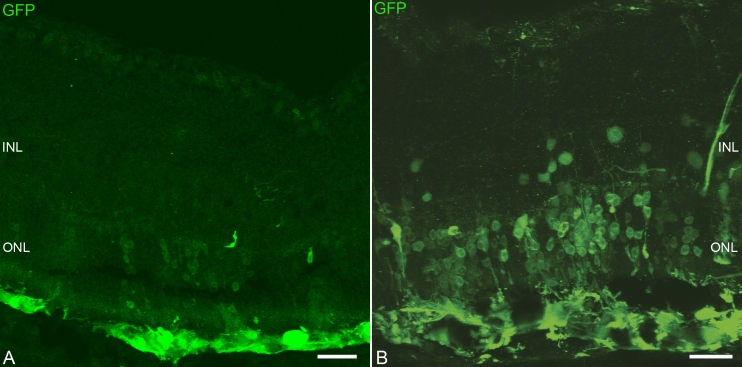
Migration of retinal progenitor cells (RPCs) into recipient retinas. A substantial number of cells migrated into the outer nuclear layer (ONL) of recipients with laser injury 3 weeks after subretinal transplantation (**B**) compared with non-injured retina (**A**). Most of the integrated cells resided in the ONL around the retinal laser lesion. Scale bar represents 75 µm.

We sought to determine whether experimental laser-induced damage promotes migration and integration of grafted RPCs into the outer retina. Expanded RPCs (passage 5) from postnatal day 1 GFP transgenic mice were transplanted to the subretinal space of adult B6 mice. All the animals with laser photocoagulation presented increased cell migration and integration. Three weeks after transplantation, 1,158±320 cells per eye (n=9) had migrated and incorporated into the recipient ONL with laser injury (Figure 1B), compared to 328±199 cells per eye (n=9), which had integrated into the ONL of controls without laser injury (Figure 1A; p<0.01). Most of the integrated cells (92%±6%) resided in the ONL around the retinal laser lesion (Figure 1B).

**Figure 2:**
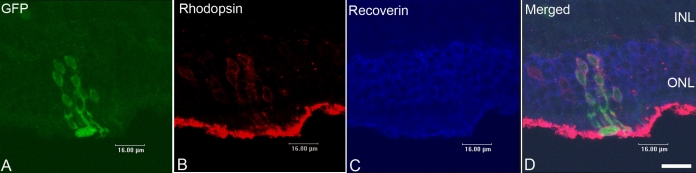
Photoreceptor identity of integrated cells. Most of the retinal progenitor cells (RPCs) integrated into the outer nuclear layer (ONL) were co-labeled with green fluorescent protein (GFP; **A**), rhodopsin (**B**), and recoverin (**C**). Panel **D** is a merged image of the three panels to the left. Scale bar represents 16 µm.

To identify the integrated cells in the ONL, immunohistochemical studies using confocal microscopy were performed 3 weeks after transplantation. Typically, rhodopsin expression was seen on the outer segments of adult mice retina. Most of the GFP+ cells (85%±11%) that had integrated into the ONL co-labeled with the photoreceptor-associated markers rhodopsin and recoverin (Figure 2). We also found that some of the grafted RPCs migrated further than the ONL. Some of those cells located in the INL seemed to express rhodopsin and recoverin. We have not characterized those cells and can not explain it at this time.

**Figure 3:**
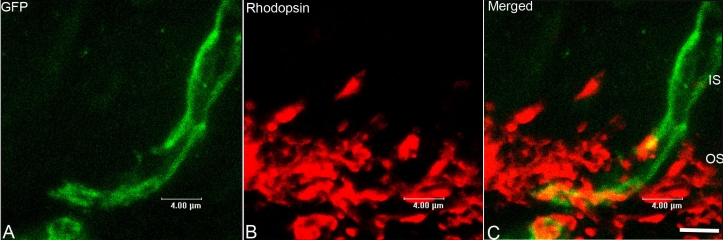
Newly developed outer segments expressed rhodopsin. Following transplantation, donor RPCs integrated into the outer nuclear layer (ONL) and developed outer segments co-labeled with green fluorescent protein (GFP; **A**), outer segment marker (rhodopsin; **B**). Panel **C** is a merged image of the two panels to the left. Scale bar represents 4 μm.

We only found that a subpopulation of integrated cells developed morphological features reminiscent of mature photoreceptors. Some of the developing outer segments expressed rhodopsin (Figure 3). We did not quantify the proportion of cells that develop these characteristics.

**Figure 4:**
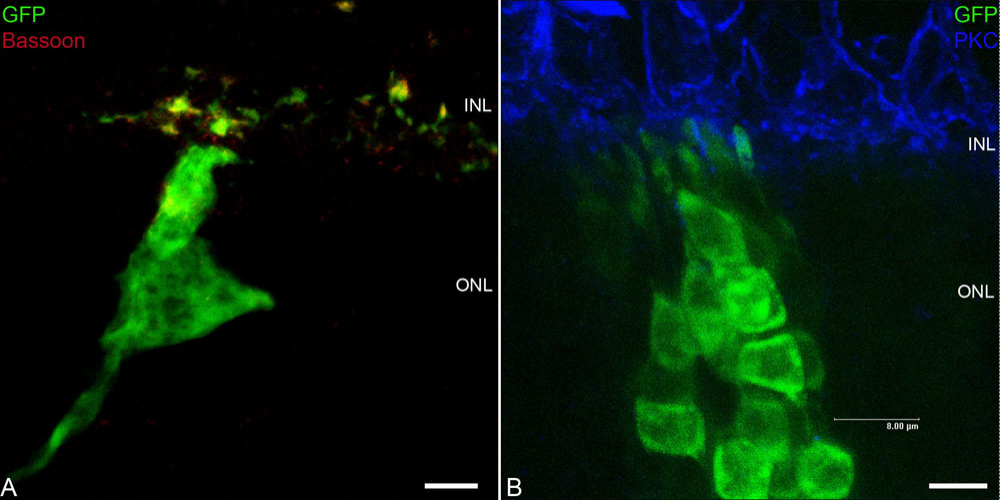
Integrated retinal progenitor cells (RPCs) expressed synaptic protein: newly developed synapses expressed the synapse protein bassoon (**A**). Synaptic connectivity of integrated cells (**B**): newly developed synapses appear to make synaptic contact with bipolar neurons, identified by immunolabeling with protein kinase C (PKC). Scale bar represents 8 µm.

To assess the synaptic connectivity of integrated cells, immunohistochemical studies using confocal microscopy were performed 3 weeks after transplantation. Integrated cells expressed the ribbon synapse protein bassoon (Figure 4A) and appear to make synaptic contact with bipolar neurons, as identified by immunolabeling with PKC (Figure 4B).

**Figure 5:**
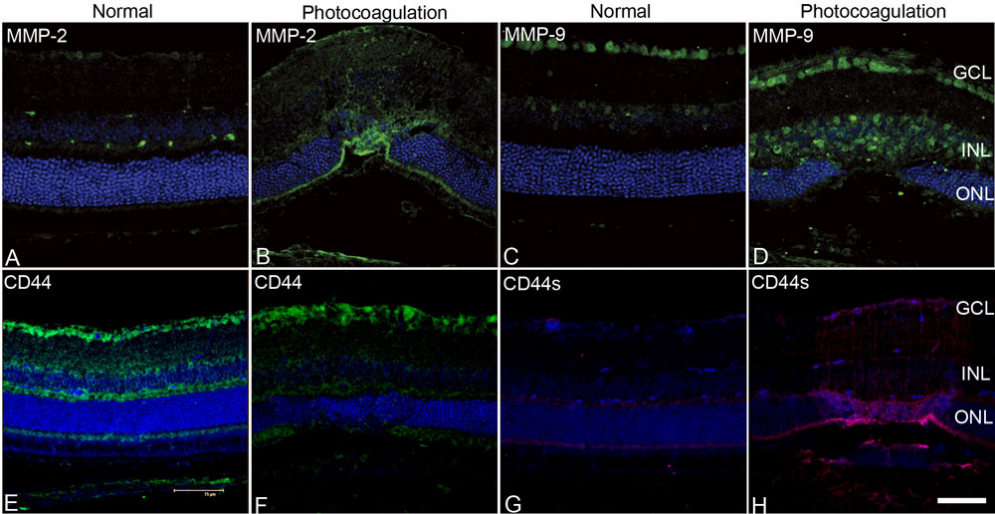
Immunoreactivities of matrix metalloproteinase-2 (MMP-2) and MMP-9. **A**-**D**: Immunohistochemical labeling of MMP-2 (**A**) and MMP-9 (**C**) in normal control retinas. Retinal photocoagulation resulted in increased MMP-2 (**B**) and MMP-9 (**D**) expression in the retinas. Immunoreactivities of cluster differentiation 44 (CD44) and CD44s. **E**–**F**: Immunohistochemical labeling of CD44 (**E**) and CD44s (**G**) in normal control retinas. Immunohistochemical labeling of CD44 in laser injured retinas (**F**). Retinal photocoagulation resulted in increased expression of CD44s (**H**), the degradation product of CD44. Nuclei are counter stained with To-Pro3. Scale bar represents 75 µm.

We next analyzed why such success is observed in this model by studying the local changes caused by retinal photocoagulation that might promote the migration of RPCs into the host retina. The typical histopathological changes associated with the laser lesions are shown in Figure 5. Injury mainly involved the outer layers of the retina.

**Figure 6:**
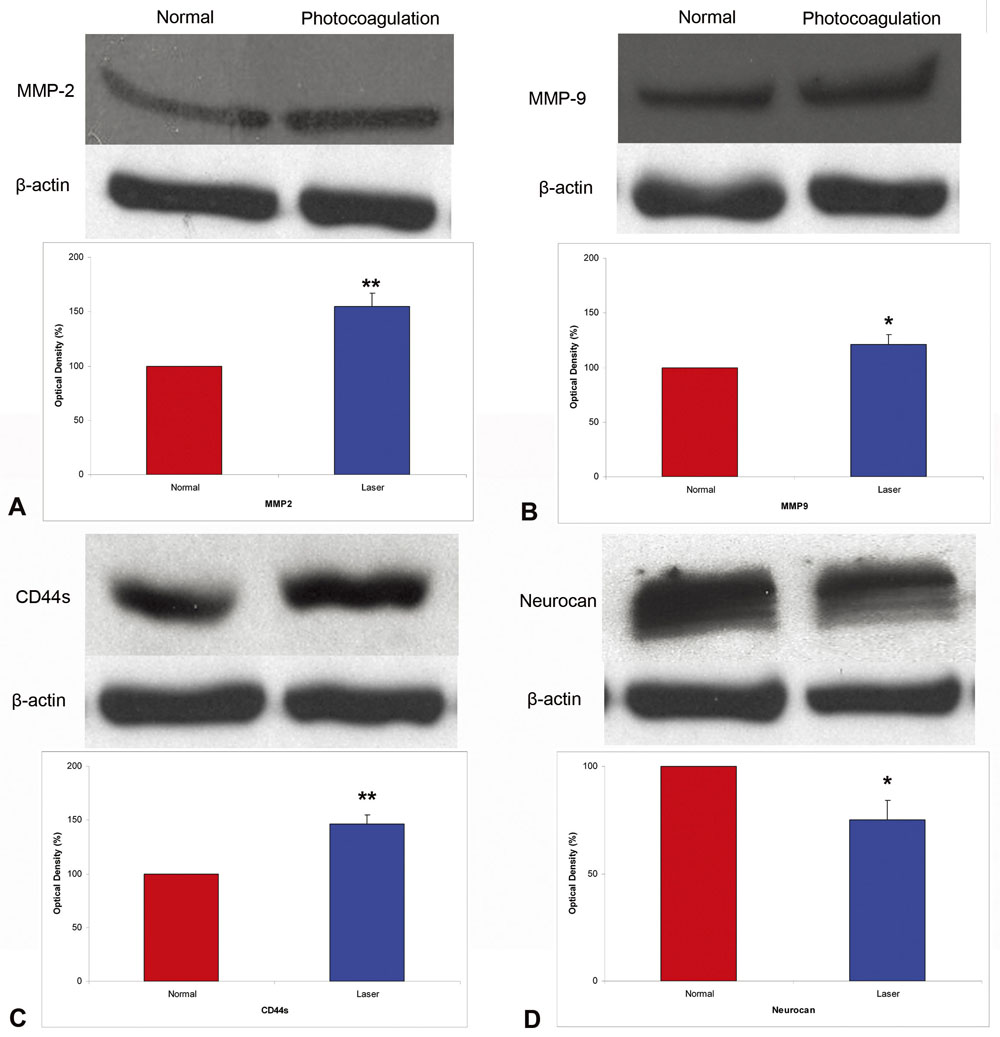
Western blot analyses. Representative western blots and corresponding densitometric analyses of matrix metalloproteinase-2 (MMP-2; **A;** n=5), MMP-9 (**B;** n=5), cluster differentiation 44 (CD44s; **C;** n=5), and neurocan (**D**). Three days after retinal photocoagulation, expression of MMP-2, MMP-9, and CD44s were significantly higher, and expression of neurocan was significantly lower, compared with that of normal control retinas. * p<0.05, ** p<0.01.

We choose to measure the levels of MMPs previously identified to promote RPC migration and integration and the levels of different extracellular proteins that have been previously identified to impede RPC migration and integration. Compared with the normal control retinas, retinal photocoagulation resulted in increased MMP-2 (Figure 5B) and MMP-9 (Figure 5D) expression in the retinas 3 days after injury (Figure 5A,C). CD44 expression in normal and laser treated retinas is shown in Figure 5E,F. Increased expression of MMP-2 and MMP-9 due to retinal laser injury was associated with increased expression of CD44s, the degradation product of CD44 (Figure 5H). Western blot analyses are shown in Figure 6. Retinal photocoagulation resulted in significantly higher levels of MMP-2 (Figure 6A, p<0.01), MMP-9 (Figure 6B, p<0.05), and CD44s (Figure 6C, p<0.01), and significantly lower levels of neurocan (Figure 6D, p<0.05), compared with normal retinas.

## Discussion

This study shows that a substantial number of transplanted RPCs, delivered by subretinal injection, migrate into the ONL of the recipient retinas with retina laser injury. In addition, a subpopulation of these cells develop morphological features reminiscent of mature photoreceptors, express photoreceptor specific proteins including synaptic protein, and appear to form synaptic connections with bipolar neurons. We demonstrate that the increased MMP-2 and MMP-9 expression seen in the retinas following laser photocoagulation is partially associated with increased migration and integration of donor RPCs from the subretinal space into the recipient ONL. This study suggests that the alteration of the extracellular matrix by a certain type of injury favors RPC migration and integration.

Migration and integration of grafted cells into diseased host tissue remains a critical challenge, particularly in the field of retinal cell transplantation. So far, stem cells transplanted into adult recipients have shown some limited evidence of being able to integrate into the ONL and differentiate into new cells expressing photoreceptor markers [4–7,29,30]. While this represents an advance over fetal tissue transplantation, it seems that natural physical barriers can also impede the migration of grafted stem cells in different CNS regions, particularly in the mature retina, in spite of the fact that immature neurons and progenitor cells are used [8–14,22,23]. Maclaren and colleagues observed that only 0.03% to 0.1% of the transplanted cells were integrated into the host retina, but the treated eyes showed improved pupillary light reflexes. The effect on the restoration of visual function would probably have been much greater if a larger percentage of the transplanted cells had been incorporated into the host retina [3,31]. Clearly, higher levels of integration of grafted cells are needed to improve or restore vision. A recent study suggests that the OLM presents a physical barrier to photoreceptor integration following transplantation into the subretinal space in the adult mice, and disruption of the OLM leads to increased retinal integration of transplanted photoreceptor precursors [24].

In a previous study of swine, we have shown that laser injury at the time of transplantation promotes retinal and RPE integration, and that the integration of donor cells is centered on lesion areas [4]. Here we show that a substantial number of RPCs (roughly 0.19% of grafted cells) migrated into the ONL of the retinas following laser injury. This result lends additional support to the concept of “wound tropism” that appears to be a hallmark of progenitor cell behavior [32,33].

In addition to the wound tropism, the significantly increased level of grafted RPCs migration into the recipient ONL seen in the present study can be attributed to the elevated MMP expression and subsequent decreased levels of inhibitory molecules, together with direct disruption of the OLM due to retinal photocoagulation. Our previous study suggests that the OLM components CD44 and neurocan inhibit cell-based retinal repair, but that elevated MMP expression leads to the proteolysis of these neurite outgrowth inhibitors [23,28]. In the present study, we have shown that MMP-2 and, to a lesser extent, MMP-9 expression increased 3 days after laser treatment, resulting in a significantly increased expression of CD44s, the degradation product of CD44, and a significantly decreased expression of neurocan. Consistent with our previous work, the altered expression of these molecules was associated with a significantly enhanced level of grafted RPC integration into the recipient ONL, compared with that of untreated controls.

We have shown that about 85% of GFP+ donor cells that migrate into the laser injured ONL express photoreceptor markers at 3 weeks following transplantation. Most notable is that a subpopulation of these cells develops morphological features reminiscent of mature photoreceptors, including the expression of the outer segment protein-rhodopsin. Some integrated cells also developed putative synapses, expressing a photoreceptor-specific presynaptic protein and appear to make synaptic contacts with host bipolar neurons.

In summary, we have shown that a substantial number of transplanted RPCs, delivered by subretinal injection, migrate into the ONL of the recipient retinas with retina laser injury. In addition, a subpopulation of these cells develop morphological features reminiscent of mature photoreceptors, express photoreceptor specific proteins including synaptic protein, and appear to form synaptic connections with bipolar neurons. We further demonstrated that the enhanced level of integration of grafted RPCs into the recipient ONL is partially associated with the increased expression of MMP-2 and, to a lesser extent, MMP-9, together with decreased levels of inhibitory molecules, following retinal photocoagulation. These results provide further evidence that the alteration of the extracellular matrix by a certain type of injury favors RPC migration and integration, and cell replacement may be a feasible strategy for treating retinal degenerative diseases.
